# Program of Intensive Support in Emergency Departments for Care Partners of Cognitively Impaired Patients: Protocol for a Multisite Randomized Controlled Trial

**DOI:** 10.2196/36607

**Published:** 2022-10-20

**Authors:** Joshua Chodosh, Karen Connor, Nicole Fowler, Sujuan Gao, Anthony Perkins, Corita Grudzen, Frank Messina, Michael Mangold, Jessica Smilowitz, Malaz Boustani, Soo Borson

**Affiliations:** 1 Division of Geriatrics and Palliative Care Department of Medicine New York University Grossman School of Medicine New York, NY United States; 2 David Geffen School of Medicine University of California Los Angeles Los Angeles, CA United States; 3 Center for Aging Research Regenstrief Institute, Inc Indiana University School of Medicine Indianapolis, IN United States; 4 Indiana University School of Medicine Indianapolis, IN United States; 5 Department of Emergency Medicine New York University Grossman School of Medicine New York, NY United States; 6 Ezkenazi Health Indianapolis, IN United States; 7 Irving Medical Center Columbia University New York, NY United States; 8 Department of Family Medicine University of Southern California Keck School of Medicine Los Angeles, CA United States

**Keywords:** emergency department, cognitive impairment, dementia, care management, root cause analysis

## Abstract

**Background:**

Older adults with cognitive impairment have more emergency department visits and 30-day readmissions and are more likely to die after visiting the emergency department than people without cognitive impairment. Emergency department providers frequently do not identify cognitive impairment. Use of cognitive screening tools, along with better understanding of root causes for emergency department visits, could equip health care teams with the knowledge needed to develop individually tailored care management strategies for post–emergency department care. By identifying and directly addressing patients’ and informal caregivers’ (or care partners’) psychosocial and health care needs, such strategies could reduce the need for repeat acute care. We have used the terms “caregiver” and “care partner” interchangeably.

**Objective:**

We aimed to describe the protocol for a randomized controlled trial of a new care management intervention, the Program of Intensive Support in Emergency Departments for Care Partners of Cognitively Impaired Patients (POISED) trial, compared with usual care. We described the research design, intervention, outcome measures, data collection techniques, and analysis plans.

**Methods:**

Emergency department patients who were aged ≥75 years and screened positive for cognitive impairment via either the Mini-Cog or the proxy-reported Short Informant Questionnaire on Cognitive Decline in the Elderly, with a planned discharge to home, were recruited to participate with their identified informal (family or friend) caregiver in the 2-site POISED randomized controlled trial at New York University Langone Health and Indiana University. The intervention group received 6 months of care management from the POISED Care Team of registered nurses and specialty-trained paraprofessionals, who perform root cause analyses, administer standardized assessments, provide advice, recommend appropriate referrals, and, when applicable, implement dementia-specific comorbid condition protocols. The control group received care as recommended at emergency department discharge (usual care) and were given information about resources for further cognitive assessment. The primary outcome is repeat emergency department use; secondary outcomes include caregiver activation for patient health care management, caregiver depression, anxiety, and experience of social support as important predisposing and time-varying enabling and need characteristics. Data were collected from questionnaires and patients’ electronic health records.

**Results:**

Recruitment was conducted between March 2018 and May 2021. Study findings will be published in peer-reviewed journals and presented to peer audiences, decision makers, stakeholders, and other interested persons.

**Conclusions:**

The POISED intervention is a promising approach to tailoring care management based on root causes for emergency department admission of patients with cognitive impairment with the aim of reducing readmissions. This trial will provide insights for caregivers and emergency department and primary care providers on appropriate, personalized, and proactive treatment plans for older adults with cognitive impairment. The findings will be relevant to audiences concerned with quality of life for individuals with cognitive impairment and their caregivers.

**Trial Registration:**

ClinicalTrials.gov NCT03325608; https://clinicaltrials.gov/ct2/show/NCT03325608

**International Registered Report Identifier (IRRID):**

DERR1-10.2196/36607

## Introduction

### Background

An emergency department visit can be a “sentinel event” for an older adult; the need for urgent attention may signal a potentially serious new problem or failure in managing a chronic condition [1-3]. Although the presenting symptoms and medically related precipitation of acute decompensation are the focus for emergency department care, emergency departments do not typically deploy standardized strategies to uncover or address psychosocial and environmental underlying conditions, or patients’ unmet needs or care process precipitants, including lifestyle or care factors that may be root causes of the need for acute care in older people. These root causes can include unrecognized cognitive impairment (often caused by Alzheimer disease or Alzheimer disease–related disorders) that increases the risk or impact of an injury or illness, unmet needs of caregivers, or an unsatisfactory home situation. We use the term, “caregiver” for someone (family or friend) we assume is giving care and “care partner” for someone (family or friend) where we do not make that assumption. Older adults visit emergency departments more frequently than younger adults and are more likely to experience adverse events after discharge [4]. They tend to present with greater acuity and clinical complexity, remain longer in the emergency department, and require more care coordination at discharge [5]. Older adults are also at greater risk of readmission and death after emergency department discharge; 20% of the older adults are readmitted and approximately 20% die within the first 3 months [6-8]. Risks are magnified by cognitive impairment in addition to age-related influences on symptom presentation and multiple comorbid conditions [9], polypharmacy [10], and higher risk of adverse drug events with increasing age [11,12]; presenting complaints and history may be vague or distorted by its effects on thinking, memory, and communication. As most emergency department physicians have little geriatric training, they may lack the expertise and skills to deal with these challenges and feel less comfortable caring for older than younger patients [13], increasing the potential for diagnostic errors and ineffective or inappropriate discharge plans [6,14]. Typical emergency department discharge plans do not account for the clinical complexity that is common in this patient group or organize effective follow-up for conditions not perceived as directly related to the visit, nor do they specifically address cognitive impairment or the help caregivers may need. Attention to recognizing and mitigating the risk of repeat emergency department visits is also not usual practice, and widely adopted care transition programs developed by Coleman [15] and Naylor [16] have not been adapted specifically for cognitively impaired patients or for the emergency department. Although some emergency department discharge approaches, such as follow-up phone calls, have had some success, they lack specificity for cognitively impaired individuals, cover only brief periods of follow-up [17], and do not target root causes [18].

Cognitive impairment is present in an estimated 25% to 40% of older patients in the emergency department [19-22] and is grossly underrecognized [20] as it is in primary care practice [23,24]. Affected individuals are at risk for poor disease management and accidental injuries that require acute care, and multiple comorbid conditions are common. In a study of emergency department use at Eskenazi Health (a site for this study), people with dementia had the highest mean number of comorbidities (mean 8, SD 2.5) of any patient subgroup, and their emergency department presentation was likely to be conditioned by this complexity [25]. In addition, caregivers may use the emergency department as a source of respite or general medical care that would be better provided in a different setting [26]. Importantly, emergency department use by people with cognitive impairment can signal unmet needs for treating ambulatory sensitive conditions or continuous medical management, for coaching or support for their caregivers at home, or for deployment of community-based services to sustain care in the home setting. Emergency departments are typically not equipped to identify reasons for visits owing to cognitive impairment or unmet caregiver needs. Therefore, identifying cognitive impairment in the emergency department, coupled with planned postdischarge care management of associated medical and lifestyle risks, is clinically important and could mitigate the need for future emergency department care.

Most daily care needs of people with clinically significant cognitive impairment living in the community are provided for by unpaid caregivers, usually family or friends [27]. Caregivers report more emotional [28] and physical stress, more hours per week spent providing care, less time for themselves and other families, and had more work-related problems than caregivers of persons with noncognitive (physical) impairments [29]. In addition, emergency department use is associated with caregiver depression and care recipient functional, behavioral, and psychological symptoms [29,30]. Concerns about the needs of caregivers of persons with cognitive impairment, including dementia, have motivated the development of dementia care management programs [31,32], adult day centers, and community support services for people living with dementia and their caregivers. However, such programs do not focus specifically on high-risk or high-need patients, may not engage caregivers in health care management or even see them as partners with clinicians in managing overall medical care, or recognize the need for emergency department or other acute care as a possible signal of unmet needs. Although the patient is in the emergency department, purposeful identification of caregivers, who play indispensable roles in carrying out postdischarge care plans and assuring follow-up once the patient leaves the acute care setting, can occur only if cognitive impairment is first detected.

Our research team includes experienced investigators (JC, SB, NRF, KIC, CRG, and MAB) who have conducted trials of dementia detection and implemented dementia-specific collaborative care management programs. Our team also includes emergency department providers and international experts in psychosocial support for dementia caregivers and demonstrated an association between caregiver support and long-term reductions in nursing home placement [33]. We apply a method initially developed outside of health care—root cause analysis—to uncover potentially remediable but unrecognized factors that lead to emergency department visits. The goal is to inform and implement preventive, person-centered care strategies that may reduce the need for further emergency department care. This intervention is the first of its kind to determine the root causes of emergency department visits for patients who screen positive for cognitive impairment and provide tailored support for caregivers and patients (dyads) in post–emergency department care. In this study, we report the protocol for a randomized controlled trial of the care management intervention, Program of Intensive Support in Emergency Departments for Care Partners of Cognitively Impaired Patients (POISED) trial. POISED is the first caregiver-based intervention to look for and intervene on hidden factors that may contribute to emergency department visits among older people living with cognitive impairment.

### Objectives

We aim to report the protocol for the design, implementation, and evaluation of a care management intervention, POISED, compared with usual care. The specific aims of this study are as follows:

1. To test whether the POISED intervention will reduce patients’ recurrent acute care use over 6 months when compared with post–emergency department care for dyads not receiving POISED. We hypothesize that rates of recurrent acute care use over 6 months will be lower for patients whose family caregivers participate in POISED.
2. To test whether the 6-month POISED intervention will activate family care partners to improve management of care recipients’ health care at 3 and 6 months compared with post–emergency department care without POISED. We hypothesize that POISED will increase family caregiver activation in managing the health care of patients.
3. To test whether POISED will improve care partner psychosocial outcomes compared with post–emergency department care without POISED at 3 and 6 months. We hypothesize that caregiver depression, anxiety, and experience of social support will improve more for caregivers who are enrolled in POISED than for those referred to other care management programs.

## Methods

### Trial Design

This is a 2-site single-blind randomized clinical trial. The randomized trial of the POISED intervention is conducted in 2 cities, New York City, New York, and Indianapolis, Indiana, and in the emergency departments of their respective academic institutions: New York University Langone Health and Indiana University Health and Eskenazi Health. Both emergency departments are academic teaching facilities used to participate in clinical trials. Both have multiple sites, which are in predominantly urban and racially diverse environments. Although these were the locations for recruitment, study procedures following consent occurred outside of the emergency department environment.

The 6-month intervention is led by the POISED Care Team, consisting of a registered nurse (the care manager) and a specially trained paraprofessional (care manager associate), who administer structured assessments and perform root cause analysis. These initial assessments are used to prepare the care management team with a comprehensive understanding of the problems leading to the emergency department visits and are the basis for creating a personalized, structured dyadic outpatient care management plan to address the needs of both patients and caregivers. The intervention is delivered via telephone or in person.

### Participants

Patients in the emergency department, at New York University Langone Health and Indiana University Health and Eskenazi Health, aged ≥75 years, who screened positive for cognitive impairment and had a planned discharge to home, were recruited to participate with their identified informal care partner. Potential participants were excluded if they lacked consent capacity and had no identified care partner, or were likely to be admitted to an inpatient service. Care partners were those persons who self-identified or were identified by the patient in the emergency department as the person most likely to assist with day-to-day activities if needed. Care partners had to (1) be English- or Spanish-speaking, (2) be able to speak by telephone, (3) have adequate hearing, (4) be at least 21 years old, and (5) demonstrate capacity to consent. If they did not meet all 5 criteria, they were excluded.

### Procedure

This noninvasive health service intervention included cognitive screening administered by research staff at any point during their emergency department admission, which was inclusive of being in an observation unit. Each potential participant was screened with one of two screening tools: the Mini-Cog [34], for patients who could be interviewed directly, or the Short Informant Questionnaire on Cognitive Decline in the Elderly [35-37], given to their family care partner. A score of ≤3/5 on the Mini-Cog [38,39] or >3.4 on the Short Informant Questionnaire on Cognitive Decline in the Elderly (for those who could not complete the Mini-Cog [35-37]) was used to identify impairment. After identifying patients with probable cognitive impairment, the research staff approached them and offered participation to them and their care partners. When feasible, the research staff then conducted a baseline assessment in the emergency department or scheduled a baseline telephone interview with the care partner as soon as possible after discharge (preferably within 48 hours). Patients and care partners (dyads) were the unit of randomization to either POISED or usual care. Follow-up assessment surveys will occur at 3 and 6 months after baseline.

### The POISED Model

#### Overview

The POISED model is designed to maximize effectiveness, improve clinical outcomes, enhance family caregivers’ skills in both self-management and health care management for the care recipient (ie, both members of the dyad), and to maximize their coping behaviors. The model is designed to promote reproducibility and has 3 main overlapping phases: the initial assessment phase, the collaborative care plan development phase, and ongoing collaborative care management.

#### Conceptual Framework

**Figure 1:**
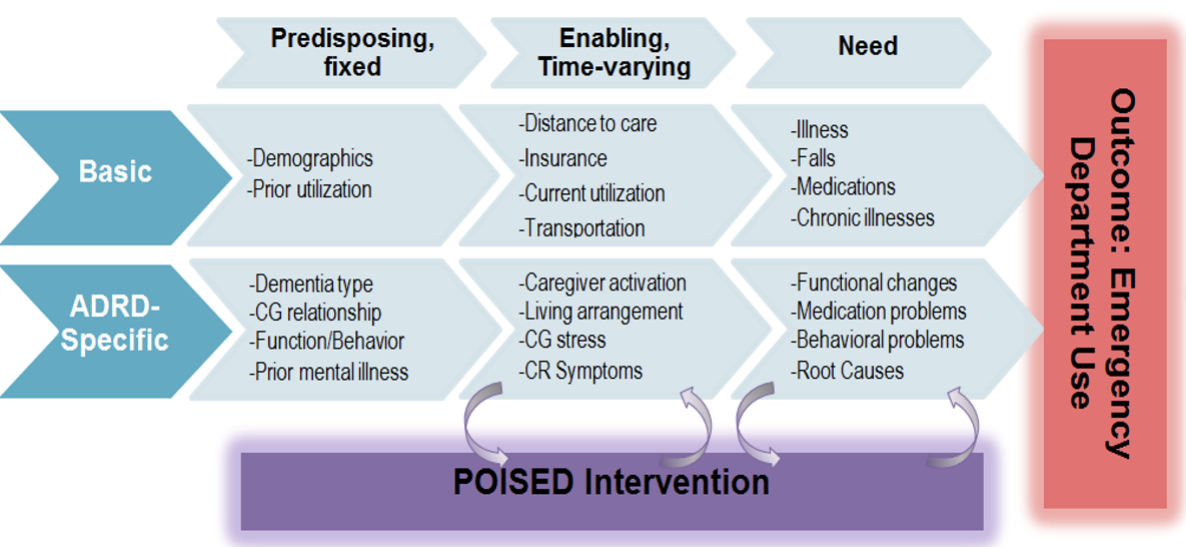
Conceptual model. ADRD: Alzheimer disease/Alzheimer Disease–Related Disorders; CG: caregiver; CR: care recipient; POISED: Program of Intensive Support in Emergency Departments for Care Partners of Cognitively Impaired Patients. Please refer to Table 1 for a more detailed list of measured factors within each category.

The POISED intervention was adapted from the Behavioral Model for Vulnerable Populations [40] originating from the Andersen behavioral model of health service use [41]. We reframed the “vulnerable” domain characteristics (Figure 1) as those particularly relevant to individuals with cognitive impairment that may substantially affect service use. These include *predisposing factors,* such as the care partner relationship (eg, spouse, child, or friend) with the care recipient, type and severity of cognitive impairment, and behavioral complications; *enabling factors*, such as care partner activation for health care management of the care recipient, in-home support resources, adequacy of the caregiving social network of family and friends); and *need factors*, such as specific treatment related to the care recipient’s acute health conditions or lack of access to medication. The POISED intervention targets these basic factors and dementia-specific factors, including knowledge about dementia and other chronic comorbid conditions, social networks (mobilizing family and friends) and support, and perceptions about health states. Dementia-specific characteristics we targeted included care partner psychological factors, effective patient and care partner self-management skills (the foundation of optimal management of chronic diseases), health system navigation skills, and health perceptions.

#### Core Strategies: Basis for Program Components

The POISED model components were motivated by dementia care quality standards as developed by the American Medical Association Physician Consortium for Quality Practice-convened Dementia Measures Work Group [42] and supplemented with chronic disease management approaches primarily directed to family caregivers. POISED components were derived from several core strategies essential for successful care management: (1) building connection and trust with dyads to increase engagement; (2) using a shared decision-making framework to establish goals that make sense to care partners, care managers, and paraprofessional care manager associates; (3) using structured, validated assessments that are comprehensive but brief enough to limit care partner burden and increase scalability for future translation; (4) increasing “on-demand” care partner access to program resources; (5) ensuring flexibility in relationships with primary care physicians in establishing allocation of responsibility; and (6) ensuring minimum standards of expertise that cover the range of biopsychosocial needs of dyads through the use of interdisciplinary care management teams.

#### Collaborative Management of Dementia Care

The burden of dementia can be reduced with collaborative management of dementia care. In 2002, Boustani et al [43] at Indiana University conducted a randomized controlled trial to compare the efficacy of a collaborative care management program for persons with dementia with the efficacy of augmented usual care. Using the chronic care model framework [44,45], this program used guideline-based biopsychosocial interventions for patients with dementia and their family caregivers (dyads). Intervention patients had significantly fewer behavioral symptoms and showed a significant 18-month improvement in depression [43]. A care management intervention, Alzheimer’s Disease Collaborative Care for San Diego Seniors, adapted a similar chronic care model framework to deliver a collaborative dementia care program within primary care and reduced dementia burden. On the basis of the mean percentage of per-patient guideline adherence, care quality for intervention group participants was better across 21 of the 23 guidelines; more dyads received community agency assistance, including respite care, than dyads in usual care [31,32]. In addition, a 3-year pragmatic cluster randomized telephone-only administered dementia care management trial within a Medicare Advantage plan improved care quality and caregiver confidence [46]. These collaborative dementia care programs, with similar chronic care model core components, were able to improve the quality of care, quality of life, and the behavioral and psychological symptoms of dementia among primary care patients and their family caregivers. The model continues to be spread in health systems for caregivers of patients already recognized as having dementia [47-49].

#### Transitioning From Acute to Postacute Settings

The Care Transitions Intervention, tested and implemented in >750 health care organizations in 40 states across the United States [50], was developed to assist vulnerable older adults in transitioning from acute to postacute settings and support increasing the capacity of patients and family caregivers in self-managing their care needs [50-52]. The Care Transitions Intervention focuses on “Four Pillars” of patient self-management: (1) medication management, (2) use of a personal health record, (3) appropriate medical follow-up, and (4) knowing how to identify and respond to a “red flag” indicative of a worsening chronic condition. We applied these strategies to the POISED intervention.

#### Preparation and Function of Care Manager Associates

Using paraprofessionals who are educated to support care management activities drives workforce development and ensures the scalability of the POISED model. Boustani et al [49] translated the collaborative dementia and depression care model into the Aging Brain Care Home funded by the Centers for Medicare and Medicaid Services Innovation Center to serve older adults in a safety net hospital system using care manager associate paraprofessionals educated in older adult care and screened for “caring.” Care manager associates are required to possess specific attributes to be capable of delivering excellent care: the ability to express caring, compassion, and empathy to both care partners and care recipients [53].

### POISED Intervention Description

The 6-month POISED care management intervention includes up to 2 in-person home visits between the dyad and the care manager and care manager associate (POISED Care Team), after an in-emergency department or phone call assessment and during the first 6 weeks after enrollment, supplemented by weekly care manager associate calls for the first month, twice-monthly calls during the second and third months, and monthly calls for the following 3 months. Although home visits are preferred, dyads remain in the intervention group even if all contacts are telephonic. Additional phone calls are scheduled with the care manager or care manager associate, as needed. A personalized dyadic care plan, “Our Action Plan,” is prepared and placed in a Care Partner Notebook (3-ring health care binder) with appropriate disease-specific infographic educational materials to teach skills for managing the dyad’s health needs on a day-to-day basis. “Our Action Plan” is a collaborative list of the care partner’s priority problems or goals (in care partner’s own words), titles of infographics, and next steps for the care partner and care manager to do. Care planning steps within the POISED disease-specific infographic educational sheets are as follows: (1) assess further; (2) inform by providing materials and education; (3) teach problem-solving and self-management or self-care; (4) make a clinical referral or follow-up appointment with a clinician; and (5) offer resources within New York University Langone Health or Indiana University Health and Eskenazi Health, or community social services in their respective regions. For clarity and usefulness, we prepared colorful infographics about disease-specific management designed to reflect the experience of caring for a person with cognitive impairment and written at an eighth grade reading level. The notebook also includes an “Introduction Letter,” “POISED Care Team Contact Sheet,” and other documents such as log sheets and an Advance Directive form, as appropriate. Supervision for the POISED Care Team is provided in twice-monthly team videoconferences with experienced dementia specialist clinicians who may help clarify root causes and make other care management suggestions. Although POISED is not embedded within the primary care context, contact is made with the patient’s primary care provider when necessary (eg, to share information relevant to medical treatment).

### Intervention Group

#### POISED Initial Assessment Phase

The POISED Care Team is structured to maximize the skill sets of specialty-trained nurses functioning as care managers and paraprofessionals in the role of care manager associates in a collaborative model. The care manager and care manager associate conduct a biopsychosocial needs assessment by phone within 48 hours of emergency department discharge if not possible during the emergency department stay. This assessment includes a demographic and psychosocial interview focused on achieving problem identification. The program uses standardized assessment tools including “Managing Your Loved One’s Health” for chronic disease management [54]. The care manager’s interview also uses principles of root cause analysis to better understand the events and potential causes leading to the emergency department visit.

The POISED Care Team documents the initial and follow-up visits, focuses on problem clarification, and reviews the assessment findings, medical records, medication lists, emergency department discharge plans, and pharmacist consultation. The care manager also reviews any diagnostic testing, any brain imaging results, and functional details of the assessment to determine the presence or absence of a likely dementia diagnosis, identifying any reversible and comorbid conditions and, for complex cases, the need for referral for further evaluation at either New York University Langone Health’s or Indiana University Health’s or Eskenazi Health’s well-developed dementia assessment centers. After reviewing the findings from prior data and the first encounter, the care manager and care manager associate create an initial plan and identify areas needing further assessment at the first home visit (within 2 weeks after enrollment). This visit enables the POISED Care Team to conduct additional cognitive and functional testing. In addition, the care manager uses the time to address more sensitive issues that the care partner may be uncomfortable discussing in the presence of the care recipient.

#### POISED Collaborative Care Plan Development Phase

This phase starts with the emergency department visit and concludes with the second home visit by the POISED care team. The goal of this phase is to create an individualized care plan through the lens of cognitive impairment, which aligns with the goals and capacities of the family care partner and care recipient. After the initial assessment is completed, any urgent issues are addressed, including consulting with the program geriatrician and primary care physician as needed. The POISED care team maps out a proposed care plan and schedules a second home visit to review the findings, discuss identified problems, and propose a collaborative plan of culturally sensitive interventions. During the second home visit, this team reviews the identified problem list, seeks input from the dyad on the continuing relevance of this list, and prioritizes the most important problems. From this consensus, the care manager discusses a proposed plan of care and customizes interventions to the dyad; explains the diagnosis, natural history, and the prognosis of dementia as necessary; and implements medical care protocols and connects patients and care partners to in-home services and community resources as needed. The POISED care team prepares and mails a personalized Care Partner 3-ring binder and follows up with a phone call to explain the binder and review materials with the care partner and patient, if able and appropriate.

#### POISED Follow-up Phase

This phase continues throughout the 6-month intervention or until the dyad is discharged from the program for reasons stipulated (see the *Criteria for Discharge From the POISED Intervention* section). During the follow-up phase, the POISED care team (primarily the care manager associate) continues to interact with the dyad by telephone, by video, by email, by fax, by mail, or face to face at their home, demonstrating a commitment to patient-centered care. The minimum amount of care manager associate contact during this time is weekly for the first month, twice-monthly calls during the second and third months, and monthly calls for the final 3 months. Interaction intensity is dictated by presenting needs and circumstances but is set at this minimum to reflect an anticipated high level of need. During these interactions, the care manager or care manager associate answers questions generated from previous visits, collects care recipient and care partner feedback, has the care partner complete a brief assessment to identify the need for specific care protocols, and facilitates care partner participation in community services already available in either the New York or Indianapolis areas. The care manager reconciles medication and reviews medication adherence at the initial and second home visit. Medication questions are referred to the study pharmacist. Throughout the follow-up phase, the team continues to work with the dyad, with contact with the patient’s primary care provider as needed, to monitor, implement, and adjust the individualized care plan. Referrals are made to local support programs that include caregiver support groups and respite care.

#### Root Cause Analysis of the Index Emergency Department Visit

Both care manager and care manager associate members of the POISED care team are well-versed in root cause analysis strategies. Starting with the emergency department visit and working backward in time, the team explores and identifies problems or branch-point situations that progress to the need for emergency department care. Using a logic tree as a cause-and-effect approach to create a timeline of events leading to emergency department visits [55] and asking the question, “How could this occur?” or “Why?” based on the “five whys” strategy [56], the POISED care team asks “why” for each successive answer, starting with “Why did you come to the emergency department?” Answers are applied to medical record review looking for other possible triggers and opportunities for intervention.

#### POISED Care Team Support

Videoconference calls among the POISED care teams are regularly scheduled (weekly to biweekly) during the study period. These calls include at least one physician specializing in dementia care and a dementia nurse specialist to offer guidance on root cause analysis discussions of patients’ emergency department visits. The root cause analysis review is structured across a 5-part domain dementia care model: (1) *body* or medical problems, (2) *behavior* or mental, (3) *brain* or cognition, (4) *buddy* or caregiver, and (5) *bank* or financial and social capital and resources. These calls also focus on ideas and methods to (1) involve the care partner and patient (as able) in goal setting and making plans to live as well as possible and (2) maximize collaboration to tailor patient-centered interventions to increase success for the care partner and patient throughout the 6-month intervention and the future. Another purpose for the video calls is to serve as an important collegial support mechanism within and across the New York University and Indiana University–based POISED care teams.

#### Criteria for Discharge From the POISED Intervention

The POISED care team used the following discharge criteria: (1) death of the patient, (2) patient or care partner declines to continue in the program, (3) primary care provider requests patient to be discharged from the program, (4) patient transitions to another health care system, (5) living situation or environment becomes unsafe for patient or care partner and therefore requires long-term skilled nursing home care, or (6) dyad completes the 6-month study.

### Control Group

The comparison group are participants randomized to usual care. They receive referrals to services at the time of enrollment. The usual care group does not receive the POISED structured assessment, root cause analysis, or associated strategies for managing chronic disease in individuals with cognitive impairment. As for intervention dyads, usual care dyads receive a laminated card showing the stress thermometer [57] to be tracked in follow-up interview assessments. Usual care dyads might be referred to care management programs at New York University and Indiana University, but such programs are not focused on issues leading to emergency department care. Moreover, our experience is that wait times for engagement in these programs can take ≥2 months and should not reduce our ability to detect between-group differences should some control group participants become engaged in an alternate form of care management support.

### Primary Outcome Measure

To assess for potential influence on any emergency department use after the index emergency department visit (primary outcome), we will use electronic medical records within each study site and identify all emergency department visits for 12 months before the index emergency department visit or study enrollment and 12 months after enrollment. We will also search all-payer databases in New York and the Indiana State Network for Patient Care (a fully operational Health Information Exchange) to identify any episode of ambulatory or acute care that occurred within the 12 months before and 12 months after enrollment (6-month intervention and 6-month follow-up). Finally, a one-item survey question will identify any additional visits that might have occurred outside the indicated state regions. For descriptive purposes, the International Classification of Diseases discharge diagnoses will be included for any emergency department use. We will structure continuous variables that describe the number of ambulatory or acute care episodes.

### Secondary Outcome Measures

#### Specific Aim 2 Measures—Caregiver Activation in Health Care Management of Care Recipient

After consenting the care partner, we will measure caregiver activation, a multidimensional construct developed by Borson et al [54] that includes caregiver knowledge, skills, and confidence to manage a range of tasks and tackle challenges common to dementia health care management [54]. For what may be the central mediating influence on emergency department use within the domain of “need,” this tool is a 29-item instrument with excellent internal consistency (Cronbach α=.95); good test-retest reliability, *r*=0.76; a strong factor structure; and strong construct validity by Rasch analysis. Domains include recognizing, anticipating, and managing day-to-day symptoms and challenges for care recipient health; managing care recipient medications; recognizing and managing sudden changes in care recipient health; accessing health services and advocating for the care recipient in the health care space; and managing caregiver self-care. Four-level item responses range from “agree completely” to “disagree completely” with an additional option, “not my job.” We will use the total score to measure activation.

#### Specific Aim 3 Measures—Psychosocial States as Important Predisposing and Time-Varying Enabling and Need Characteristics

These include caregiver depression, anxiety, experience of social support, and stress. We will use the Patient Health Questionnaire–9 (PHQ-9; [58,59]) and the 7-item Generalized Anxiety Disorder (GAD-7) scale [60,61] to determine the impact of the POISED intervention on caregivers’ mood and anxiety at baseline, 3 months, and 6 months. We have used both instruments in multiple research studies, including our dementia collaborative care trials [43,62,63]. The PHQ-9 is a 9-item depression scale with a total score from 0 to 27, and the GAD-7 is a 7-item anxiety scale with a total score from 0 to 21. Both scales have good internal consistency and test-retest reliability, as well as convergent, construct, criterion, procedural, and factorial validity for the diagnosis of major depression and general anxiety disorder [58-61]. Experience of social support will be measured using the Medical Outcomes Study (MOS) Social Support Survey—Abbreviated [64]. This is a 4-item survey that uses a 5-point Likert scale. Respondents are asked how often each kind of support is available if needed. The 4 elements of support are “someone to get together with for relaxation” (companionate support), “someone to help with daily chores if you were sick” (instrumental support), “someone to turn to for suggestions about how to deal with a personal problem” (informational with support), and “someone to love and make you feel wanted” (emotional support). Analyses comparing the abbreviated version to the original version have shown strong similarities in performance and good psychometric properties based on confirmatory factor analyses [65]. The stress thermometer [57], a visual thermometer with a 5-level analog scale to indicate the level of stress chosen by the caregiver, measures caregiver stress. Every enrolled care partner will be given a laminated card with the stress thermometer at the time of emergency department discharge for use during interviews. Lost cards will be replaced. We will use the Healthy Aging Brain Care (HABC) monitor to adjust for dementia symptom severity [66]. The HABC monitor is a caregiver survey tool for monitoring 3 care recipient symptom domains (cognitive, functional, and behavioral or psychological) and a caregiver quality of life measure. It has good internal consistency (0.73-0.92) and construct validity compared with the Neuropsychiatric Inventory [67], and is sensitive to 3-month change compared with Neuropsychiatric Inventory “reliable change” groups [66].

### Other Outcome Measures

Additional outcome measures (Table 1), in addition to primary and secondary measures, cover predisposing, enabling, and need characteristics and serve as important covariables within the regression model testing emergency department use as the primary outcome.

**Table 1:** Predisposing, enabling, and need characteristics.

Measure				Data source		Measure construction		Measures refer to CG^a^ or CR^b^; assessment times: 0 or BL^c^, 3, 6, and 12 months
**Predisposing fixed characteristics**								
	**Basic**							
		Age	EHR^d^ or survey		Years		CG, CR; 0 months (BL)	
		Sex	EHR or survey		Categorical: Male or female		CG, CR; 0 months (BL)	
		Race or ethnicity	EHR or survey		Categorical: White, Black, Hispanic, other		CG, CR; 0 months (BL)	
		Education	Survey		Categorical: <High school, high school, some college, college graduate+		CG; 0 months (BL)	
		Prior (1-year) nonacute use	EHR or survey		Counts: Physician ambulatory visits		CR; 0 months (BL)	
		Prior (1-year) acute use	EHR or survey		Counts: ED^e^, hospital visits or bed days		CR; 0 months (BL)	
	**AD/ADRD^f^ specific**							
		Dementia type	EHR		Categorical: Including Alzheimer disease, Lewy Body disease, Parkinson disease, vascular, frontotemporal, and mixed		CR; 0, 3, and 6 months	
		Caregiver relationship to CR	Survey		Categorical: Spouse, child, other relative, friend or other		CG, CR; 0 months	
		Functional state	Survey		14 items: Activities of Daily Living or Instrumental Activities of Daily Living [68] for CG; within HABC-M^g^ [66] for CR		CG, CR; 0, 3, and 6 months	
		Marital status	Survey		Categorical: Single or never married, married, divorced, widowed		CG, CR; 0, 3, and 6 months	
		Substance use history	Survey		Current: Yes or no; past history: yes or no		CG, CR; 0, 3, and 6 months	
		Mental illness history	Survey		Yes or no: depression, schizophrenia, posttraumatic stress disorder, other		CG, CR; 0 months	
**Enabling time-varying effect characteristics**								
	**Basic**							
		Distance to hospital (ED)	Calculated		Miles		CG, CR; 0, 3, and 6 months	
		Distance to usual source of care (USC)	Calculated		Miles		CG, CR; 0, 3, and 6 months	
		Difference in distance ED vs USC	Calculated		Miles (USC)—distance to hospital		CG, CR; 0, 3, and 6 months	
		Change in PCP^h^	Survey		Yes or no		CR; 0, 3, and 6 months	
		Insurance	EHR		Yes or no		CR; 0, 3, and 6 months	
		Current nonacute use	EHR or survey		Counts: physician ambulatory visits, in-home supportive services		CR; 0, 3, 6, and 12 months	
		Current acute use	EHR or survey		Counts: physician ambulatory visits, ED, hospital visits or bed days		CR; 0, 3, 6, and 12 months	
	**AD/ADRD specific**							
		Mode of transportation	Survey		Categorical: personal car, taxi, train, bus, walk		CR; 0, 3, and 6 months	
		Caregiver living arrangement	Survey		Categorical: Live with subject, close proximity (miles), other		CG; 0, 3, and 6 months	
		Caregiver stress	Survey		Stress thermometer (scale), 5-level visual analog scale [57]		CG; 0, 3, 6, and 12 months	
		Dementia symptoms	Survey		HABC-M [66] (measuring severity of CR symptoms)		CR; 0, 3, and 6 months	
**Need characteristics**								
	**Basic**							
		Acute illness	Survey		Counts: episodes by type (classification by study physicians)		CR; 0, 3, and 6 months	
		Falls, other injuries	Survey		Counts: episodes by type (classification by study physicians)		CR; 0, 3, and 6 months	
		Nonacute illness	Survey		Counts: episodes by type (classification by study physicians)		CR; 0, 3, and 6 months	
		Medication need for refill	Survey		Categorical: Yes or no		CR; 0, 3, and 6 months	
		Clinical comorbid conditions	EHR		Charlson comorbidity index [69]		CR; 0, 3, and 6 months	
		Satisfaction	Survey		Scale: 0-10 (worst possible care to best possible care [70])		CG; 0, 3, and 6 months	
	**AD/ADRD specific**							
		Functional state	Survey		14 items: Activities of Daily Living or Instrumental Activities of Daily Living for CG [68]; within HABC-M [66] for CR		CG, CR; 0, 3, and 6 months	
		Caregiver activation	Survey		MYLOH^i^ Instrument [71]		CR; 0, 3, 6, and 12 months	
		Social support	Survey		MOS^j^ Abbreviated Social Support (4-item; 5-point Likert scale [64,72])		CG; 0, 3, and 6 months	
		Other root causes	EHR		Application of post hoc adjudication of root causes for ED use		CR; 0, 3, and 6 months	

^a^CG: caregiver.

^b^CR: care recipient.

^c^BL: baseline.

^d^EHR: electronic health record.

^e^ED: emergency department.

^f^AD/ADRD: Alzheimer disease/Alzheimer Disease–Related Disorders.

^g^HABC-M: Healthy Aging Brain Care Monitor.

^h^PCP: primary care physician.

^i^MYLOH: Managing Your Loved One’s Health.

^j^MOS: Medical Outcomes Study.

### Sample Size Calculations

**Figure 2:**
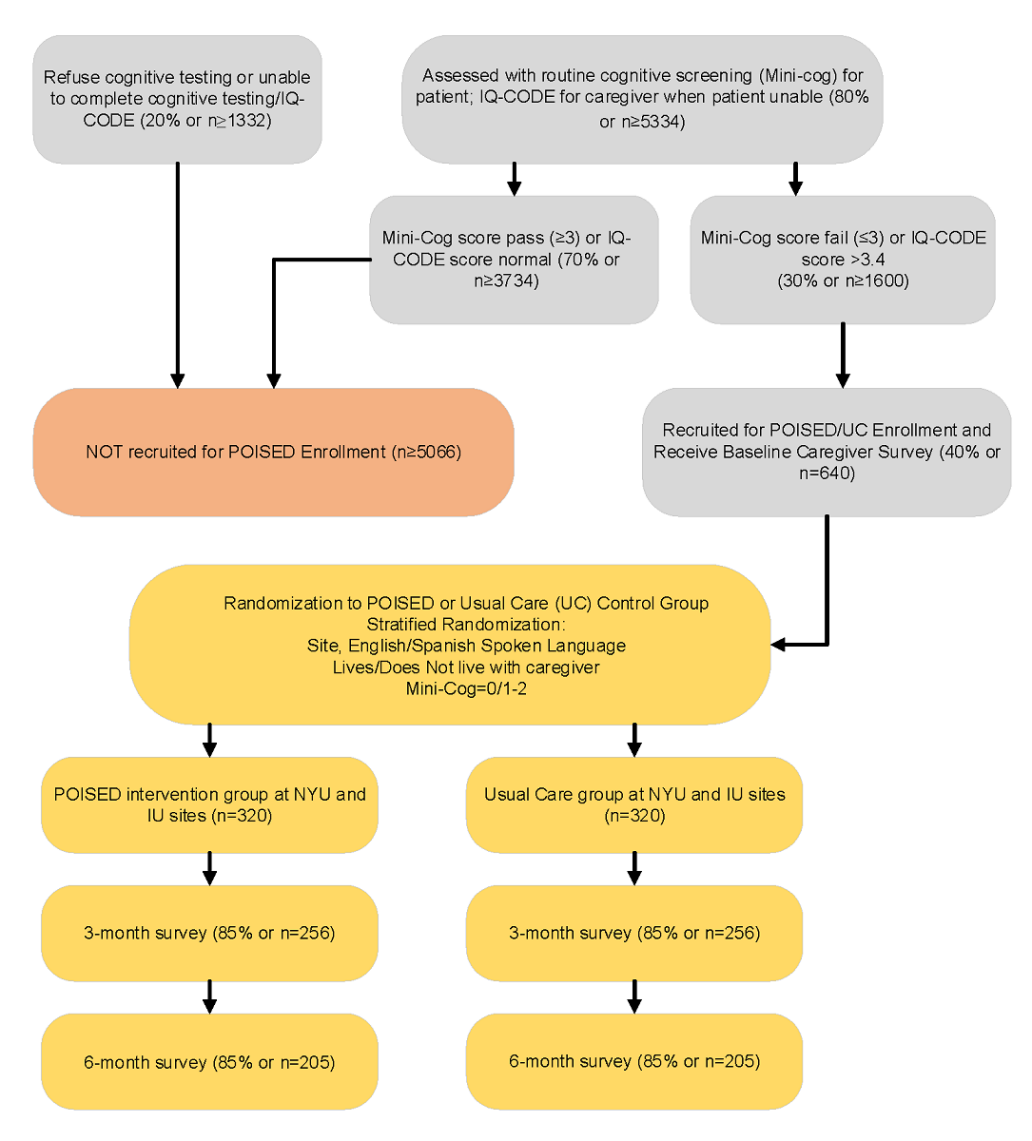
Recruitment or enrollment flowchart. IQ-CODE: Informant Questionnaire on Cognitive Decline in the Elderly; IU: Indiana University; NYU: New York University; POISED: Program of Intensive Support in Emergency Departments for Care Partners of Cognitively Impaired Patients.

Using conservative prevalence statistics for cognitive impairment in patients presenting to the emergency department at New York University Langone Health, Indiana University Health, and Eskenazi Health, numbers (6100 patients in 2015) exceed those needed (Figure 2) to achieve 80% power for detecting reduction in acute care use in the POISED group compared with the usual care group in specific aim 1. A previous study reported a 30-day readmission rate of 58% in patients with dementia compared with 38% in those without dementia [26]. Given that our patient sample may include individuals with less severe cognitive impairment and less emergency department use, we assume, conservatively, that the rate of repeat emergency department visits in the usual care group is 40%. With 320 patients enrolled per group, we will have 82% power to detect an odds ratio of 0.62 for repeat emergency department visits in the POISED group compared with the usual care group at the 0.05 significance level. This detectable odds ratio is equivalent to reducing the emergency department visit rate to 29% or lower in the POISED group compared with the 40% assumed for the control group. The use of stratified randomization reduces the variance of the difference between the 2 group means and results in greater power than simple randomization [73,74]. Therefore, the actual power for our study will be higher than that projected here. As we will be using electronic health records for acute care data and phone follow-up to supplement out-of-network use, we anticipate complete data from all study participants for this aim.

For aims 2 and 3, assuming that 205 (64%) dyads will complete the 6-month evaluation (Figure 2), we will have 80% power to detect an effect size ≥0.28 on the caregiver activation score, PHQ-9, GAD-7, and MOS scores between the POISED group and the control group using a 2-tailed *t* test at a .05 significance level. The detectable effect size of 0.28 used in our power estimation is reasonable and justified given that previous studies on collaborative care management of dementia patients have shown an effect size of 0.45 SD on a number of caregiver psychosocial outcomes [43]. Previous studies have found a mean PHQ-9 score of 4.4 (SD 5.6) and a mean GAD-7 score of 3.2 (SD 3.5 [43,58,59,61]). Thus, our projected effect size will allow us to detect a change as small as 1.6 on the PHQ-9 and 1 on the GAD-7. As described earlier, the use of stratified randomization will provide greater power than that projected here.

### Randomization

To produce comparable groups and insure against accidental bias in treatment assignments, we used a computer-generated web-based stratified randomization scheme. Dyads were randomized to intervention or usual care groups in random blocks of 4 or 6 stratified by site (New York University Langone Health or Indiana University Health and Eskenazi Health).

### Statistical Methods or Analysis Plan

#### Overview

We will compare randomization results to the preplanned schedule to ensure randomization integrity. To verify the comparability of the randomized groups, we will compare dyads between the 2 groups to identify differences in their baseline characteristics (age, sex, race, and education), care recipient comorbid medical conditions, and the Charlson comorbidity index [69,75]. We will also look for differences in the number of primary care visits and acute care use during the year before enrollment between the POISED group and the usual care group by using analysis of covariance models for continuous variables and the Cochran-Mantel-Hansel statistic for categorical variables controlling for the stratification variable of recruitment site. We will examine the distributions of continuous variables and use transformation or nonparametric methods in cases of violation to the normal distribution assumption. We will also examine the frequency distribution of all categorical variables and use exact inference procedures in cases of 0 or small cell size. We will use SAS (version 9.4; SAS Institute) for all the analyses.

#### Specific Aim 1

We will use logistic regression models to compare the rates of emergency department admission during the 6-month intervention period following the index (recruitment) emergency department visit. Emergency department readmission within 6 months will be used as a binary outcome in the logistic model, and the randomization group will be the independent variable while adjusting for site. Baseline characteristics that are shown to be unbalanced in univariate comparisons between the 2 groups will also be adjusted. Although not a primary outcome, we will also examine 12-month emergency department use data.

#### Specific Aim 2

We will use mixed effects models with caregiver activation scores at 3 months and 6 months as the outcome measure and randomization group as the independent variable while controlling for baseline activation score and site. We will conduct post hoc comparisons of the activation scores between the POISED group and the usual care group at 3 and 6 months using linear contrast from the mixed effects model following a significant group effect. To explore what changes are responsive to the POISED intervention, we will also use the mixed effects model to examine differences in activation domain scores between the 2 groups. The mixed effects model will account for potential correlations between repeated measures from the same individual and deal with missing data appropriately when the probability of missing data is unrelated to the missing observation.

#### Specific Aim 3

We will use mixed effects models with repeatedly measured PHQ-9, GAD-7, and MOS social support scores collected at 3 and 6 months as dependent variables. The independent variable for the mixed effects model will be the indicator variable for the randomization group while controlling for baseline scores and site. We will use post hoc analysis to determine group differences in these measures between the 2 groups at the 3- or 6-month evaluations. The modeling approach resembles that for aim 2.

#### Sensitivity Analysis for Missing Data

The analysis plan outlined above assumes that outcome measures at follow-up are missing at random with respect to demographic characteristics and baseline results. We will compare baseline characteristics of participants with missing outcomes because of death or withdrawal to detect potential violations of the missing-at-random assumption. Further sensitivity analyses will involve various imputation methods or a full parametric likelihood approach that assumes various patterns of missing data [58].

### Data Monitoring

To proactively maintain high quality in data collection, data are routinely checked as they are stored within study databases. Data are quality-checked before analysis.

#### Data Collection

Data will come from 3 sources. First, the web-based tracking system of data entered by the POISED Care Team will use a REDCap (Research Electronic Data Capture; Vanderbilt University) database [76]. These data will provide us with extensive information on the process and content of care for those randomized to the POISED. This system is designed to support and monitor clinical care delivered by the POISED Care Team. We have fields to support the POISED intervention care processes, including results of assessment instruments, content of the tailored intervention, and clinical observations such as dyads’ level of participation. Second, we will obtain data on health services use including all diagnostic testing and medication use and use of inpatient and outpatient services from 1 year before study enrollment to 1 year after enrollment (2-year duration) from the New York University Langone Health’s Epic system and the Indiana Network for Patient Care [77]. Data are obtained from electronic medical records by a team of data managers employed by New York University (DataCore) and the Regenstrief Institute in support of clinical research. Third, the primary outcome measure data will come from telephone interviews and are entered in a separate REDCap database. A research interviewer collects complete telephone survey data from caregivers at baseline, 3 months, and 6 months using a 30-minute survey, blinded to randomization assignment. Care manager associates collect much briefer 12-month follow-up data (6 months after the intervention) from intervention and control caregivers as a measure of treatment effect sustainability. The interviewer enters deidentified survey data from each survey wave into a Health Insurance Portability and Accountability Act–compliant REDCap electronic database hosted at New York University [76]. REDCap baseline data are available to the POISED Care Team so that specific relevant data can inform care manager previsit data and limit redundancy in questions and caregiver interview burden.

#### Data Management

DataCore, a resource launched by the New York University Langone School of Medicine, housed within its Information Technology Department and formed in collaboration with the Clinical and Translational Science Institute, the Biomedical Informatics and Translational Library Programs, and the Department of Population Health, will provide enterprise level support to ensure the integrity of electronic data during its capture, storage, management, extraction, and sharing. DataCore will merge these 3 data streams using unique identifiers assigned to the study participants and provide regular backups onto a secure server.

### Ethical Considerations and Data Confidentiality

The Institutional Review Boards of New York University Langone Health and Indiana University Health provided permission on February 24, 2017, and April 27, 2017, respectively, to conduct the POISED study (approval number: i16-01473_CR2). The clinical trial registration (NCT03325608) was listed on October 30, 2017. At enrollment, we obtained consent from patients (or assent with care partner proxy consent when the patient lacked the capacity to consent) to enable the review of their medical records. The proxy consent was the next of kin using a defined hierarchy in the absence of a Durable Power of Attorney or by consenting the proxy who is the Durable Power of Attorney. Participants completed an “education session” during the consent process regarding the potential risks and benefits of participating in a noninvasive health services research study for valid informed consent. Electronic data are stored in password-protected files on secure servers at New York University and Indiana University. Research staff are trained in the institutional review board–approved methods for collecting, recording, storing, and reporting research data to protect the privacy of participants and maintain the confidentiality of data.

## Results

Data collection began in March 2018 and was completed in February 2022. We have recruited 643 dyads (patients and care partners). Data are currently being analyzed. The study results will be published in peer-reviewed journals and presented to peer conferences, decision makers at the participating university medical centers, and other interested audiences.

## Discussion

### Potential Impact

Studying care management for older adults living with cognitive impairment and possible undetected dementia who are identified at emergency department visits may lead to low-cost strategies to reduce high-cost acute care. It may also improve caregivers’ ability to optimally assist patients in the management of chronic disease that is particularly challenging in older adults with cognitive impairment. Related impacts may be improved health outcomes of patients and psychosocial well-being of caregivers.

### Limitations (Threats to Validity)

The most significant limitation will be the dependence on one care management team at each site, as findings may reflect the personal qualities of the care manager and care manager associate. However, a clearly communicated and structured intervention protocol and standardized education and training program will limit the quality and reproducibility concerns. We may find no change in caregiver activation while demonstrating reductions in acute care use. An absence of change (aim 2) may reflect an inadequate measure or a factor that is not in the pathway of health service use. However, the opportunity to demonstrate a causal relationship between activation and use for this population is profoundly important. Given that usual care participants may receive dementia-specific resources given well-developed programs at both institutions, the comparative between-group differences might be diminished. However, engagement for usual care participants is not likely to occur for at least 2 months after the index emergency department visit and will not be focused on management strategies that reflect an applied root cause analysis for that visit. Although some emergency department patients will have a diagnosis of Alzheimer disease or Alzheimer disease–related dementia before their emergency department visit, some patients may not have Alzheimer disease or related dementia (even after a positive screen) and generalizability to future Alzheimer disease or related dementia patients might be questioned. However, the cognitive screener and older age group for inclusion should limit false positives, and diagnostic errors should be equivalent across groups.

### Dissemination

Cognitive impairment is prevalent among older adults who visit the emergency department, but often this is unrecognized. Root cause analysis linked to care management strategies may better focus the intervention resulting in more reductions in acute care use while more directly addressing care partners’ needs. This study may provide effective low-cost approaches for reducing high-cost acute care use and related improvements in care partners’ abilities to optimize management of chronic diseases particularly challenging for care recipients with cognitive impairment. Moreover, this highly standardized and reproducible approach has the potential for direct application in large multisite clinical intervention trials. The use of paraprofessionals as care management assistants increases the scalability of this work and provides opportunities for large-scale implementation and dissemination if proven efficacious.

### Conclusions

The POISED program is a promising approach to address the root causes for emergency department admission in older adults with cognitive impairment and prevent repeated readmissions. The results from this trial will provide insights for care partners and medical staff on proactive treatment plans with appropriate and personalized management plans for these older adults and for care partners themselves. These findings will be relevant to both professionals and nonprofessionals concerned with the quality of life for individuals with cognitive problems and their care partners.

## Abbreviations

GAD-7: 7-item Generalized Anxiety Disorder
HABC: Healthy Aging Brain Care
MOS: Medical Outcomes Study
PHQ-9: Patient Health Questionnaire–9
POISED: Program of Intensive Support in Emergency Departments for Care Partners of Cognitively Impaired Patients
REDCap: Research Electronic Data Capture

## Data Availability

Data will be available upon request after the analyses are completed and the data are properly deidentified.
